# Investigating the Interplay between Nucleoid-Associated Proteins, DNA Curvature, and CRISPR Elements Using Comparative Genomics

**DOI:** 10.1371/journal.pone.0090940

**Published:** 2014-03-03

**Authors:** Hao Tong, Jan Mrázek

**Affiliations:** 1 Department of Statistics, University of Georgia, Athens, Georgia, United States of America; 2 Department of Microbiology and Institute of Bioinformatics, University of Georgia, Athens, Georgia, United States of America; University of Rome Tor Vergata, Italy

## Abstract

Many prokaryotic and eukaryotic genomes feature a characteristic periodic signal in distribution of short runs of A or T (A-tracts) phased with the DNA helical period of ∼10–11 bp. Such periodic spacing of A-tracts has been associated with intrinsic DNA curvature. In eukaryotes, this periodicity is a major component of the nucleosome positioning signal but its physiological role in prokaryotes is not clear. One hypothesis centers on possible role of intrinsic DNA bends in nucleoid compaction. We use comparative genomics to investigate possible relationship between the A-tract periodicity and nucleoid-associated proteins in prokaryotes. We found that genomes with DNA-bridging proteins tend to exhibit stronger A-tract periodicity, presumably indicative of more prevalent intrinsic DNA curvature. A weaker relationship was detected for nucleoid-associated proteins that do not form DNA bridges. We consider these results an indication that intrinsic DNA curvature acts collaboratively with DNA-bridging proteins in maintaining the compact structure of the nucleoid, and that previously observed differences among prokaryotic genomes in terms DNA curvature-related sequence periodicity may reflect differences in nucleoid organization. We subsequently investigated the relationship between A-tract periodicity and presence of CRISPR elements and we found that genomes with CRISPR tend to have stronger A-tract periodicity. This result is consistent with our earlier hypothesis that extensive A-tract periodicity could help protect the chromosome against integration of prophages, possibly due to its role in compaction of the nucleoid.

## Introduction

Bacterial nucleoid is organized in dynamic supercoiled loops [1], [2]. Although this model of nucleoid structure is based on studies of a few model organisms, it appears likely that this type of chromosome organization is widespread and possibly universal among bacteria. The specific organization of the DNA loops is largely determined by interactions of the DNA with nucleoid-associated proteins (NAPs) [1], [3]. The nucleoid conformation is related to gene expression and the dynamic character of NAP-DNA interactions facilitates regulation of the nucleoid structure. For example, the *E. coli* nucleoid organization differs in exponential and stationary growth phase [1]. However, it was proposed that DNA intrinsic curvature, that is, DNA bending encoded in the nucleotide sequence and independent of interactions with proteins or other molecules, also affects the structure of the nucleoid [4]. The intrinsic DNA curvature is primarily caused by clustered A-tracts, or short runs of A or T in the DNA sequence, periodically spaced in phase with the DNA helical period of about 10.5 bp.

DNA curvature-related A-tract periodicity is widespread among both prokaryotes and eukaryotes [5], [6], [7]. However, the exact role of the intrinsic DNA curvature in prokaryotic chromosomes is not clear. Curved DNA segments are often associated with promoters [8], [9] and could also reflect the predominant mode of DNA supercoiling [5], [10]. In this work, we focus on the hypothesis that the intrinsic bends contribute to the compaction of the DNA in the nucleoid [4]. Our recent analysis of more than 1000 prokaryotic chromosomes revealed significant differences among different organisms in the overall intensity of the periodic signal as well as intrachromosomal variance of the A-tract periodicity. In most genomes, strong A-tract periodicity, indicative of extensive DNA curvature, is restricted to segments covering relatively small fraction of the chromosome. In contrast, some genomes feature a strong A-tract periodicity that persists over majority of the chromosome length [6]. We also noted that in the genomes with persistent strong A-tract periodicity, highly expressed genes tend to localize in the intervening segments of low periodicity. Based on these results, we proposed that the prokaryotic nucleoid consists of segments that are structurally more rigid and characterized by strong A-tract periodicity indicative of high content of intrinsically curved DNA alternating with structurally more flexible regions characterized by weak A-tract periodicity and low content of curved DNA [6]. In a subsequent work, we noted that prokaryotic chromosomes with persistent A-tract periodicity are less likely to contain prophages compared to chromosomes that lack persistent A-track periodicity [11]. This observation is consistent with our notion that the A-track periodicity and the associated DNA curvature relate to local rigidity of the nucleoid structure, which hinders processes such as transcription or integration of foreign DNA.

If DNA curvature and the concomitant A-tract periodicity contribute to nucleoid compaction one might expect to find a relationship between the A-tract periodicity and the properties of NAPs in different species. Consequently, the primary goal of this work was to use comparative genomics to investigate the interplay between intrinsic DNA curvature and NAPs. In particular, we were interested in finding whether differences between genomes in terms of the overall A-tract periodicity were related to the ensemble of NAPs used by different organisms. Pursuant to the finding that A-tract periodicity may contribute to protection of the genome from phage integration [11], we also investigated the relationship between the A-tract periodicity and presence of CRISPR (Clustered Regularly Interspaced Short Palindromic Repeats), which have been implicated in protection against phages [12].

## Methods

### Quantitative Measure of A-tract Periodicity (MaxQ Index)

Intrinsic DNA curvature is largely associated with periodically spaced A-tracts. In this work, we used two different definitions of A-tracts, namely dinucleotides AA and TT (A2T2 method) and tetranucleotides AAAA/AAAT/AATT/ATTT/TTTT (AT4 method) [6]. These two choices of A-tract definition reflect alternative models of intrinsic DNA curvature [13], [14], [15]. The A-tract periodicity in a genome is assessed by the PerPlot algorithm [6], [16]. The algorithm starts by constructing a histogram of spacings between pairs of A-tracts, that is, counting the number of times *N*(*s*) a pair of A-tracts occur at the mutual distance *s* (measured in base pairs). The histogram is subsequently processed to reduce noise and artifacts from repetitive sequences and periodic signals unrelated to DNA curvature (e.g., the 3-bp periodicity arising from protein-coding sequences), and the final histogram is converted to a power spectrum by Fourier transform. The power spectrum is scaled to enable comparisons among sequences with distinct characteristics such as nucleotide and oligonucleotide composition. The MaxQ index is the height of the largest peak in the power spectrum [6]. A detailed description of the algorithm was presented in refs. [6], [16] and on the Web (http://www.cmbl.uga.edu/software/Perplot_HTML/Perplothtml.html). The software is available on our web site (http://www.cmbl.uga.edu/software/).

In this work, we modified the MaxQ definition to include only the largest peak in the range of periods 9.5–11.5 bp. This is equivalent to the MaxQ* that we used in a previous work [11]. The purpose of this modification is to reduce the possibility of misinterpreting peaks unrelated to DNA curvature as DNA curvature-related peaks because periodic signals with periods outside the 9.5–11.5 bp range sometimes arise from tandem repeats in the genome. For example, collagen-like repeats in the encoded proteins often generate a periodic signal with 9 bp period.

#### DNA sequences

Annotated nucleotide sequences of complete prokaryotic genomes were downloaded from the NCBI FTP server (ftp://ftp.ncbi.nih.gov/genomes/Bacteria/). To reduce statistical artifacts of duplicated observations we randomly selected only one genome per species when multiple strains of the same species were available and calculated their MaxQ* values. The final dataset included 573 organisms (File S1). For genomes consisting of multiple chromosomes the analysis was performed on the largest chromosome.

### KEGG Orthology

KEGG (Kyoto Encyclopedia of Genes and Genomes) is a bioinformatics resource for understanding higher-order functional meanings and utilities of the cell or the organism from its genome information [17]. KEGG orthology (KO) database and KEGG GENES database were used to determine which of the standard NAPs are present in each genome. We use the KEGG classification because in our experience it is more accurate in identifying truly orthologous proteins than alternative databases such as COGs [18] or OrthoDB [19], which sometimes combine functionally distinct paralogs in the same “orthologous” group. The standard collection of NAPs was adopted from ref. [1]. For NAPs that were widely distributed among the genomes in our dataset (Table 1), we first found their corresponding K numbers in KO database and then used the ‘LinkDB Search’ on KEGG website (http://www.genome.jp/linkdb/) to search by its KO number against the GENES database to obtain a full list of genomes which possess such protein.

**Table 1:** A list of nucleoid associated proteins.

K Number	Gene	DNA bridging
K05516	CbpA, curved-DNA-binding protein A	N/A
K04047	Dps, DNA protection from starvation	N/A
K03557	Fis, factor for inversion stimulation	+
K03746	H-NS, histone-like nucleoid-structuring	+
K05787	hupA, DNA-binding protein HU-alpha	N/A
K03530	hupB, DNA-binding protein HU-beta	N/A
K04764	ihfA, integration host factor alpha	N/A
K05788	ihfB, integration host factor beta	N/A
K03719	Lrp, leucine-responsive regulatory protein	+
K03632	MukB, chromosome partition protein	+
K11685	stpA, DNA-binding protein	+

K Number and Gene are the KEGG Orthology number and abbreviated name of protein; DNA bridging:+indicates that the protein has DNA bridging function in a model organism, while N/A means that the protein is not known to form DNA bridges. The classification was adopted from ref. [1].

### Statistical Analysis

Each NAP listed in Table 1 was investigated for a relationship with MaxQ* values. The dataset of 573 genomes was divided into two groups: with and without the particular NAP. The Mann-Whitney U test was used to evaluate the difference in MaxQ* indices between the two groups of genomes. This procedure was repeated for each NAP. We are concerned that some phyla that do not include well-studied model organisms may contain additional NAPs not listed in Table 1. To reduce the possibility that our results are influenced by such differences between different phyla, we performed the same analysis restricted to proteobacteria and γ-proteobacteria, which are the most represented groups in our dataset and contain many of the well-characterized bacterial species.

### Intra-chromosomal Analyses in *E. coli K12*

In addition to comparisons among different genomes we investigated intrachromosomal correlations between DNA curvature and NAP binding potential in *E. coli K12*. The binding potential of each NAP in a chromosomal region was measured by the number of its predicted binding sites in that region. The binding sites were predicted using the Motif Locator program (http://www.cmbl.uga.edu/software/motloc.html), which applies the standard position-specific score matrix (PSSM) model. A collection of known binding sites used to build the PSSM was extracted from DPInteract [20] for H-NS, Lrp, IHF and Fis. The choice of these four NAPs for this analysis was dictated by data availability. The analysis was performed with two values of score cutoffs to verify that the qualitative results do not depend on a particular setting of the score cutoff. Higher score cutoff leads to fewer positive prediction, thus improving specificity at the expense of sensitivity, whereas lower cutoff increases sensitivity at the expense of specificity.

In-house software was developed to predict intrinsic DNA bends. The program implements the method proposed by Tolstorukov and coworkers [4]. Their method is based on a simplified DNA curvature model, which assumes that A-tracts represent a dominant contribution to DNA curvature. By assigning a DNA bending angle to each A-tract and assuming that the DNA helical period is constant at 10.5 bp, one can predict the deviation of DNA helical axis over a certain window length (in bp). In this work, we used two sets of parameters for predicting bends: In the first set of parameters we define an intrinsic bend as any DNA segment no longer than 100 bp where the DNA helical axis at the start and end of the segment deviates by at least 60 degrees (designated ‘60w100 bends’); the second set of parameters detect bends of at least 45 degrees over the length of at most 60 base pairs (‘45w60 bends’). The *E. coli* genome was divided into 10 kb non-overlapping segments and we recorded the number of predicted binding sites for each of the four NAPs and the number of predicted DNA bends in each window. No correction was used for intragenomic variance in nucleotide and oligonucleotide composition, codon bias, gene content, or any other factors. Binding sites or bends that spanned two adjacent segments contributed ½ count to each segment. Correlations between the numbers of binding sites and number of bends were subsequently evaluated. The information on predicted binding sites and DNA bends is presented in File S2.

### Relationship between DNA Curvature and Presence of CRISPR Elements

The relationship between DNA periodicity and presence or absence of CRISPR elements was evaluated in a manner analogous to assessments of the relationship between DNA periodicity and NAPs. We used the information in CRISPR database [21] (as available in December 2011) to divide the 573 genomes in our data collection into two groups based on whether they contained CRISPR or not. “Questionable structures” reported in the database were not considered. The Mann-Whitney U test was used to assess significance of the difference of MaxQ* indices between the two groups.

### Relationship between NAPs and CRISPRs

In order to detect the possible relationship between NAPs and CRISPRs, we built contingency tables for each NAP and CRISPRs. Then Fisher’s exact test was performed to obtain the *p*-value for each pair. For those NAPs with significant relationship with CRISPRs, we did U tests for MaxQ* while controlling the factor of NAP presence/absence, i.e. we compared MaxQ* values between groups with and without CRISPRs, but both with and without the particular NAP.

## Results

### Relationship between Nucleoid Associated Proteins and Sequence Periodicity

All NAPs appeared to have significant relationship with the MaxQ* values at the level of p<0.05 (Table 2 and File S3). However, the *p*-value of StpA is not statistically reliable because of a highly asymmetric data sample (only 10 of the 573 genomes possess StpA). Other NAPs with less asymmetric absent/present ratios (around 10 or less) all have *p*-value less than 0.05. Notably, all bridging proteins have strong relationship with MaxQ* characterized by *p*-values <10^−3^, whereas HU-α is the only non-bridging protein with very low *p*-value. Other non-bridging NAPs have p-values >10^−3^ and mostly >0.01. Notably, all NAPs have positive correlation with MaxQ*, that is, presence of the NAP in the genome is associated with higher MaxQ*, suggesting that higher DNA curvature is generally found in genomes with more diverse NAP repertoire.

**Table 2:** Summary MaxQ* statistics in genomes having and lacking specific NAPs in all analyzed genomes.

Interaction mode	NAP	“A2T2” method				
		Genomes with the NAP		Genomes lacking the NAP		*p*-value
		N	Mean MaxQ* ± SD	N	Mean MaxQ* ± SD	
Bridging	H-NS	130	3.08±0.76	443	2.78±0.71	<10^−4^
Bridging	StpA^a^	10	3.90±0.49	563	2.83±0.72	<10^−4^
Bridging	MukB^a^	47	3.50±0.52	526	2.79±0.72	<10^−12^
Bridging	Lrp	255	3.00±0.73	318	2.72±0.72	<10^−5^
Br+Be^b^	Fis	143	3.08±0.76	430	2.77±0.71	<10^−5^
Bending	IHF-β	256	2.91±0.73	317	2.79±0.74	0.028
Bending	IHF-α	250	2.92±0.73	323	2.78±0.73	0.010
Bending	HU-α	67	3.32±0.74	506	2.78±0.71	<10^−7^
Bending	HU-β	456	2.88±0.72	117	2.70±0.78	0.006
None^c^	Dps	306	2.91±0.73	267	2.77±0.73	0.009
None^c^	CbpA	150	2.98±0.79	423	2.80±0.71	0.019

Mean MaxQ* values assessed by the “A2T2” method and standard deviations are shown for groups of genomes possessing and lacking a homolog of each NAP. N signifies the number of genomes in each group. Presence of absence of each NAP in a genome is based on data from the KEGG database ([17], http://www.genome.jp/linkdb/). ‘Interaction mode’ specifies whether the NAP forms bridges or bends upon interaction with DNA (adapted from [1]). Statistical significance of the differences was assessed by Man-Whitney U-test. Data corresponding to p-values ≥0.05 are in parentheses. DNA-bridging NAPs are in the top part of the table.

a The ratio of genomes with and without the NAP is unbalanced (>10 or <0.1).

b Fis can form both bridges and bends.

c Dps and CbpA have not been confirmed to form bridges or bends.

Table 3 and Table 4 are analogous to Table 2, but restricted to proteobacteria and γ-proteobacteria among the 573 genomes, respectively. While the statistical significance of the relationships decreases in the smaller collections of genomes, bridging proteins still exhibit significant relationship with DNA curvature and generally achieve much lower *p*-values than non-bridging proteins. We therefore conclude that DNA curvature tends to be more pronounced in genomes with larger repertoire of DNA bridging proteins. Application of the ‘AT4’ method to calculate the MaxQ* index yielded similar results (File S4).

**Table 3:** Summary MaxQ* statistics in genomes having and lacking specific NAPs in proteobacteria.

Interaction mode	NAP	“A2T2” method				
		Genomes with the NAP		Genomes lacking the NAP		*p*-value
		N	Mean MaxQ* ± SD	N	Mean MaxQ* ± SD	
Bridging	H-NS	127	3.07±0.76	143	2.83±0.69	0.02
Bridging	StpA^a^	10	3.90±0.49	260	2.91±0.71	<10^−4^
Bridging	MukB	47	3.50±0.52	223	2.83±0.72	<10^−7^
Bridging	Lrp	172	3.08±0.73	98	2.72±0.68	<10^−3^
Br+Be^b^	Fis	143	3.08±0.76	127	2.79±0.66	0.002
Bending	IHF-β	237	(2.95) ±0.71	33	(2.92) ±0.85	0.95
Bending	IHF-α	243	(2.94) ±0.72	27	(2.83) ±0.69	0.4
Bending	HU-α	67	3.32±0.74	203	2.82±0.69	<10^−5^
Bending	HU-β	244	(2.94) ±0.72	26	(2.99) ±0.81	0.69
None^c^	Dps	178	(2.99) ±0.75	92	(2.86) ±0.68	0.16
None^c^	CbpA	104	(3.02) ±0.78	166	(2.90) ±0.70	0.28

See legend to Table 2.

a The ratio of genomes with and without the NAP is unbalanced (>10 or <0.1).

b Fis can form both bridges and bends.

c Dps and CbpA have not been confirmed to form bridges or bends.

**Table 4:** Summary MaxQ* statistics in genomes having and lacking specific NAPs in γ-proteobacteria.

Interaction mode	NAP	“A2T2” method				
		Genomes with the NAP		Genomes lacking the NAP		*p*-value
		N	Mean MaxQ* ± SD	N	Mean MaxQ* ± SD	
Bridging	H-NS	77	3.39±0.73	31	2.83±0.80	0.002
Bridging	StpA	10	3.90±0.49	98	3.16±0.78	0.002
Bridging	MukB	46	3.51±0.52	62	3.02±0.89	0.005
Bridging	Lrp	90	3.38±0.73	18	2.49±0.68	<10^−4^
Br+Be^b^	Fis^a^	99	3.29±0.76	9	2.51±0.75	0.008
Bending	IHF-β^a^	100	3.29±0.76	8	2.43±0.80	0.008
Bending	IHF-α^a^	101	3.30±0.76	7	2.20±0.50	0.001
Bending	HU-α	66	(3.33) ±0.74	42	(3.07) ±0.85	0.14
Bending	HU-β	92	(3.24) ±0.78	16	(3.15) ±0.85	0.67
None^c^	Dps	75	3.35±0.77	33	2.97±0.79	0.019
None^c^	CbpA	45	(3.14) ±0.89	63	(3.29) ±0.71	0.44

See legend to Table 2.

a The ratio of genomes with and without the NAP is unbalanced (>10 or <0.1).

b Fis can form both bridges and bends.

c Dps and CbpA have not been confirmed to form bridges or bends.

**Figure 1:**
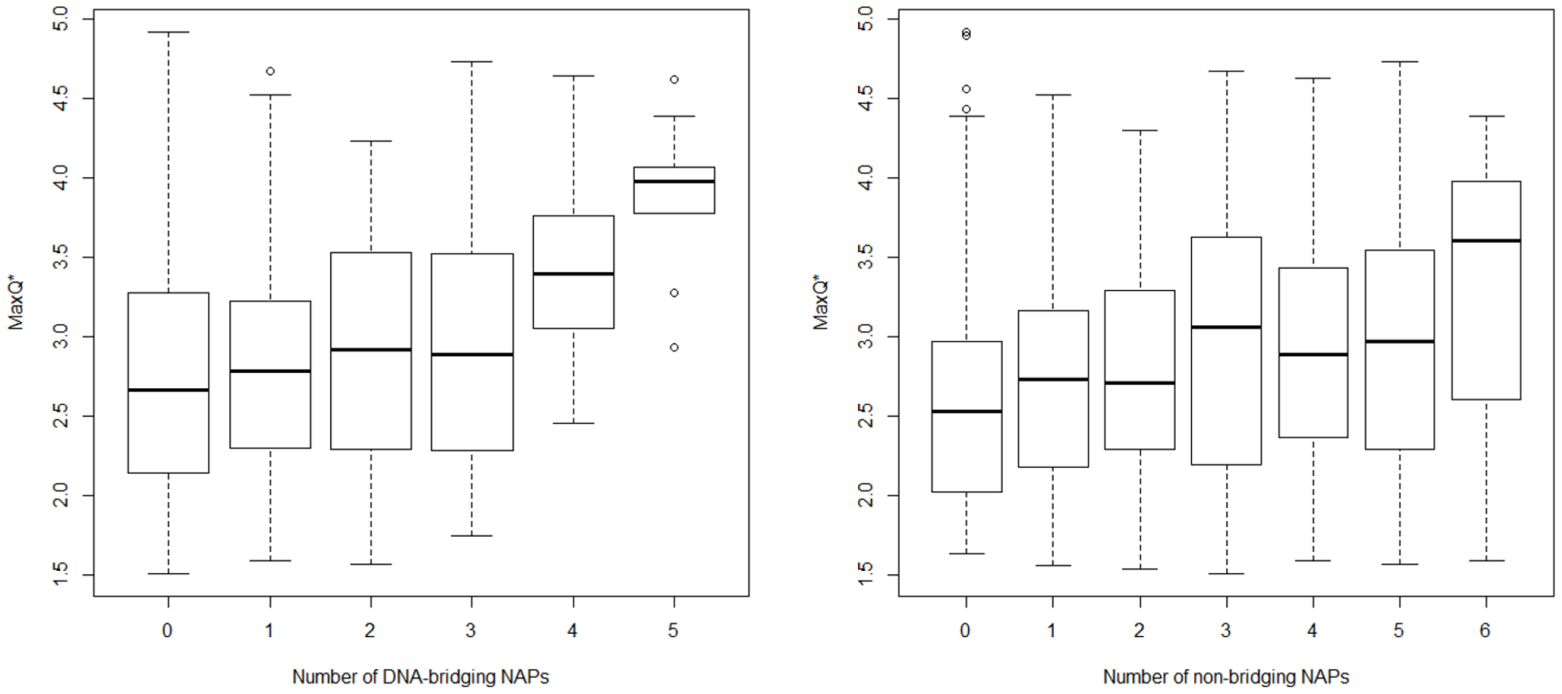
The ‘box and whiskers’ plots showing the distribution of MaxQ* values in genomes that contain different numbers of the investigated DNA-bridging (left) and non-bridging (right) NAPs. The bold bar shows the median, the box covers the range between 25^th^ and 75^th^ percentiles, and the top and bottom bars indicate the maximum and minimum excluding outliers.

Interestingly, all NAPs show positive correlations with A-tract periodicity, that is, genomes with a given NAP tend to have on average higher MaxQ* than genomes without the NAP. This is true for both bridging and bending NAPs and even if the differences have low statistical significance (Tables 2–4). We interpret this observation as an indication that genomes with more diverse repertoire of NAPs tend to have stronger A-tract periodicity and possibly higher content of intrinsically curved DNA. Figure 1 shows the distribution of MaxQ* values for genomes that contain different numbers of DNA-bridging NAPs and non-bridging NAPs. The MaxQ* values correlate positively with the number of both bridging and non-bridging NAPs while the correlation is stronger for the bridging NAPs. The Pearson correlation coefficients for bridging and non-bridging NAPs are 0.26 and 0.18, respectively. However, this conclusion could be affected to some extent by the fact that the collection of NAPs analyzed in this work is not complete and some genomes likely contain additional (possibly unknown) NAPs.

We performed the same analysis for a collection of 25 metabolic genes, which are unlikely to have a direct relationship with nucleoid structure, although there could still be an indirect relationship due to possible effects of various metabolites (e.g., reactive oxygen species) on DNA (File S5). While several of the 25 genes exhibit statistically significant relationships with MaxQ*, only few reach p-values as low as the DNA-bridging NAPs in our dataset, and only one, *moaD* encoding a molybdopterin synthase sulfur carrier subunit, shows p-values similar to the DNA-bridging NAPs across all three datasets (all genomes, proteobacteria, and γ-proteobacteria). While these results indicate that the p-values could be underestimated for some genes (possibly due to biases related to phylogenetic distribution of genes) the collection of DNA-bridging NAPs is unusual in comparison to this control set because all bridging NAPs exhibit very significant relationships with MaxQ*.

### Local NAP Binding Potential Correlates with DNA Bends in *E. coli K12*

**Figure 2:**
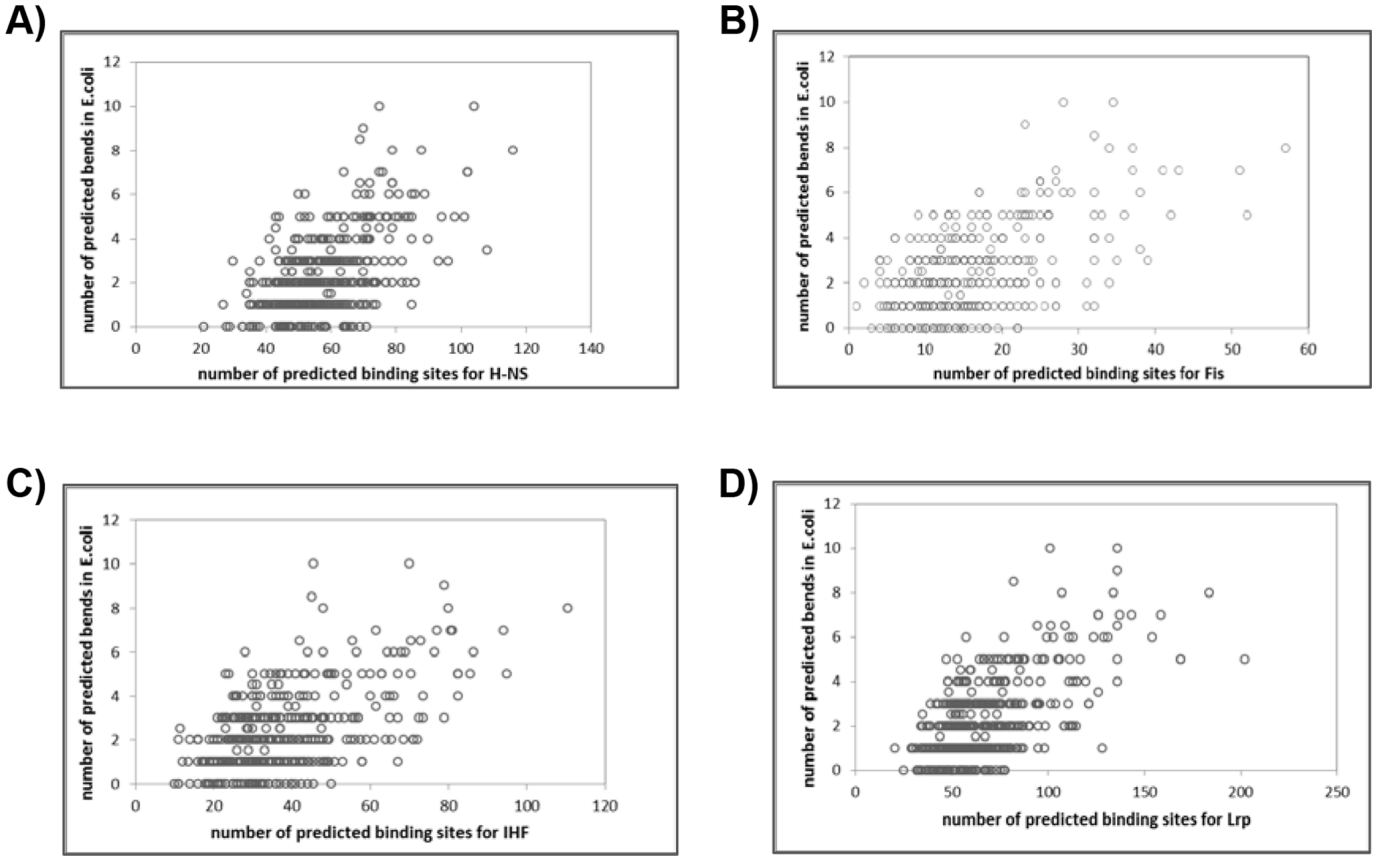
Correlation between the number of predicted DNA bends and the number of predicted binding sites in *E. coli K12* for a) H-NS, b) Fis, c) IHF and d) Lrp.

We evaluated the correlations between the number of predicted DNA bends and the number of predicted binding sites for H-NS, Fis, IHF and Lrp. The data presented here apply to the 60w100 bends and binding site predictions obtained with the default cutoff score for Motif Locator. **Figure 2** shows strong positive correlations for all four proteins. The Pearson correlation coefficients for the four proteins are 0.55, 0.55, 0.56, and 0.62, respectively. All 4 correlations are significant at *p*<0.001. Similar results were obtained with 45w60 bends and Motif Locator score cutoff increased by 3 and for *Salmonella typhimurium* LT2 genome (File S6).

### Relationship between Sequence Periodicity and CRISPR

CRISPR is a component of molecular machinery that protects bacteria against previously encountered bacteriophages using a mechanism resembling the immune system of higher eukaryotes [12]. CRISPR was found in most archaeal and many bacterial genomes, and based on our analyses, organisms containing CRISPR tend to have higher MaxQ* (U test *p*-value is 0.009), which is indicative of higher content of curved DNA.

### Relationship between NAPs and CRISPRs

Three NAPs, namely IHF-α, IHF-β and Dps, exhibit a significant relationship with CRISPR, all three with a positive trend (i.e., genomes with these NAPs are more likely to contain CRISPR; Table 5). This observation indicates that the concurrence of these NAPs and CRISPRs may further complicate the relationship between DNA curvature and CRISPRs. In order to determine if the relationship between CRISPR and MaxQ* was a consequence of the relationships between CRISPR and the three NAPs listed above we performed the Mann-Whitney U test using only genomes that contain the specific NAP (Table 6). The low *p*-values suggest that the relationship between DNA curvature and CRISPR is not a simple consequence of the relationship between the NAPs and CRISPR.

**Table 5:** Fisher exact test between NAPs and CRISPRs.

NAP	N1	N2	N3	N4	*p*-value
IHF-β	98	177	88	79	0.0005 (+)
HU-β	205	70	135	32	0.132
cbpA	73	202	41	126	0.656
stpA	7	268	0	167	0.048
MukB	27	248	9	158	0.109
Fis	61	214	40	127	0.726
Lrp	109	166	71	96	0.618
H-NS	53	222	37	130	0.543
IHF-α	95	180	86	81	0.0005 (+)
HU-α	33	242	17	150	0.643
Dps	111	164	106	61	0.000002 (+)

N1 is the number of genomes with BOTH the NAP and CRISPR; N2 is the number of genomes with ONLY CRISPR; N3 is the number of genomes with ONLY the NAP; N4 is the number of genomes with NEITHER the NAP NOR CRISPR; (+) indicates a positive correlation significant with *p*<0.01.

**Table 6:** Correlations between CRISPR and MaxQ* while controlling for the relationship between CRISPR and NAPs.

NAP	Genomes with CRISPR		Genomes lacking CRISPR		*p*-value
	N	Mean MaxQ* ± SD	N	Mean MaxQ* ± SD	
IHF-β	98	3.01±0.77	88	2.67±0.67	0.002
IHF-α	95	3.03±0.76	86	2.67±0.68	0.002
Dps	111	3.09±0.74	106	2.68±0.71	<10−4

The Mann-Whitney U test was performed using only genomes that contain the specified NAP. The analysis was performed for three NAPS that show significant relationship with CRISPR (Table 5). See legend to Table 2.

## Discussion

This work follows from a hypothesis that intrinsic DNA curvature and the associated periodic spacing of A-tracts in bacterial genomes contribute to the compaction and maintenance of the nucleoid structure [4]. We previously reported significant differences among prokaryotic genomes in terms of A-tract periodicity [6]. Because the nucleoid structure is largely determined by interactions between DNA and NAPs we expected that differences in A-tract periodicity could be related to differences in the repertoires of NAPs present in different genomes. Indeed, we detected weak but statistically significant correlations between the intensity of A-tract periodicity (measured by the MaxQ* index) and presence or absence of NAPs in different genomes. Notably, the relationship between A-tract periodicity and NAPs was strongest for DNA bridging proteins, while among non-bridging NAPs only HU-α exhibited a strong correlation with MaxQ* (Tables 2–4). This result is not necessarily surprising. The DNA bridges are formed by NAPs that bind to two or more sites in DNA, which may be distant in the sequence, and oligomerize, thus locking the binding sites in close spatial proximity [1], [3]. However, to allow the oligomerization, the binding sites first have to be brought close together, which can be facilitated by plectonemic supercoiling where intrinsic bends are likely to be positioned at the tip of the supercoiled loop to minimize the deformation stress on the DNA. Indeed, H-NS binds preferentially in the proximity of bent DNA while Fis binding sites in *E. coli* are known to contain clustered A-tracts [22], [23]. By contrast, NAPs that cause DNA bending directly at the binding site (DNA bending proteins) do not necessarily require prior intrinsic bend. Hence, our result that presence of DNA bridging NAPs in genomes correlates with stronger periodicity of A-tracts is consistent with the hypothesis that nucleoid compaction is aided by collaborative action of NAPs and intrinsically curved DNA segments. This hypothesis is further supported by the intrachromosomal correlation of the number of predicted bends and number of NAP binding sites in *E. coli* (Figure 2).

The rules governing nucleoid organization in bacterial cells are largely unknown, in part because of limited availability of appropriate experimental methods. Consequently, several important caveats should be considered in interpretation of these results. In the absence of ability to measure the amount of curved DNA directly, we rely on indirect measures based on A-tract periodicity, which are noisy and in some cases can be distorted by various forms of repeats in the genome [6]. Moreover, cellular concentrations of NAPs could differ significantly among different bacteria. Unfortunately, the relevant data on NAP concentrations are unavailable for vast majority of the organisms included in this work. Therefore, we can only consider presence or absence of a particular gene, which can be determined with a reasonable accuracy from the genome sequence. There may also be subtle nuances in the function of homologous NAPs among distinct genomes and many organisms contain additional NAPs not analyzed in this work, which might substitute for missing NAPs. Additional problems arise from redundancy of NAP functions, where one NAP can compensate for absence of another. Unfortunately, the detailed roles of individual NAPs in many diverse genomes are still poorly understood and most of the relevant information is unavailable. All these factors increase noise in our data. However, in spite of these limitations and uncertainties, we believe that interpretation of our data in support of the general hypotheses that intrinsic DNA curvature plays a role in nucleoid compaction and that difference among genomes in terms of A-tract periodicity are related to differences in NAP repertoires is justified. In particular, the stronger correlations of A-tract periodicity with DNA bridging NAPs compared to non-bridging NAPs is consistent with mechanisms how different NAPs contribute to nucleoid compaction. In addition, the consistence of the general trends among different data samples (Tables 2–4) is encouraging. While our data on NAPs in the complete collection of genomes in our dataset is incomplete, the smaller datasets restricted to proteobacterial and further to γ-proteobacteria probably contain less missing data, in part because these groups include many extensively studied model organisms (Table 7). Moreover, the collection of genomes used in this work was selected to provide a statistically large sample while reducing sampling biases that could lead to exaggerated assessments of statistical significance.

**Table 7:** NAP distribution in different phyla.

Phyla	Sample size	H-NS	Fis	Lrp	MukB	stpA	IHF-α	IHF-β	HU-α	HU-β	Dps	CbpA
γ-proteobacteria	108	71%	92%	83%	43%	9%	94%	93%	61%	85%	69%	42%
β-proteobacteria	46	80%	89%	85%			98%	98%		98%	80%	48%
α-proteobacteria	80	14%		45%			94%	88%	1%	91%	64%	3%
δ-proteobacteria	21	5%	10%	29%	5%		100%	100%		90%	19%	90%
ε-proteobacteria	15									93%	67%	100%
Cyanobacteria	10			10%						100%	80%	90%
Firmicutes	87			29%						97%	57%	7%
Actinobacteria	48			65%						83%	75%	13%
Tenericutes	21									90%	10%	
Bacteroidetes	14			36%						93%	64%	43%
Chlorobi	10	30%		100%				10%		100%		
Chloroflexi	6									83%	67%	50%
Chlamydiae	7										14%	
Thermotogae	9									100%		
Euryarchaeota	35			17%								
Crenarchaeota	17											
Spirochaetes	13						8%	62%		8%	85%	54%

Phyla represented by more than 5 genomes in our dataset are shown. Sample size is the number of genomes representing each particular phylum. Bridging NAPs are shown in bold face. The numbers in the cells indicate the percentage of genomes in the phylum that have the specific NAP, blank cell means 0.

As a secondary goal of this work, we investigated the relationship between A-tract periodicity and presence of absence of CRISPR elements. This effort stems from our previous work, which indicated that persistent DNA curvature could help protect the genome from integration of prophages [11]. CRISPR elements are involved in protection of their hosts from bacteriophages via mechanism that resembles eukaryotic immunity system [12]. We speculate that maintenance of CRISPR machinery requires a selective constraint in the form of repeated exposure to phages. Although this assumption has not been confirmed, it is possible that organisms with CRISPR are generally more frequently exposed to phages in their natural environments compared to organisms lacking CRISPR. At the same time, neither CRISPR nor A-tract periodicity provides absolute protection against phages. CRISPR functions by storing short segments of phage DNA, which subsequently serve to silence the phage DNA via a mechanism analogous to RNA interference. While the CRISPR efficiency in silencing phage genes is high it can only act on previously encountered phages and some phages are resistant to CRISPR [24]. We assume that A-tract periodicity aids chromosome compaction and decreases its conformational flexibility, which hinders but not necessarily prevents integration of foreign DNA [11]. If this is correct then the A-tract periodicity could be maintained, at least in part, by the same selective constraint imposed by repeated phage exposure that maintains CRISPR elements. This hypothesis is consistent with the result that genomes with CRISPR tend to have stronger overall A-tract periodicity than genomes lacking CRISPR. Thus, it is possible that CRISPR and extensive DNA curvature both play a role in protection against phages. However, recent reports that some NAPs are directly involved in regulation of CRISPR-associated *cas* genes in *E. coli* and *Salmonella enterica* [25], [26] point to a possibility that there could also be a direct relationship between NAPs and presence of CRISPR in the genome.

## Conclusions

We conclude that our results concerning relationships between presence of nucleoid-associated proteins and intensity of A-tract periodicity in bacterial genomes are consistent with the hypothesis that intrinsically curved DNA segments contribute to nucleoid compaction and that differences among genomes in terms of A-tract periodicity can be related to differences in nucleoid structure [4], [6]. Specifically, the intrinsic DNA bends act in conjunction with DNA-bridging NAPs to stabilize the plectonemically supercoiled DNA loops that constitute bacterial nucleoid. Moreover, the correlation of A-tract periodicity with presence of CRISPR suggests that maintenance of strong A-tract periodicity and CRISPR could be influenced by the same selective constraint, presumably in the form of repeated exposure to phages. This is consistent with our earlier hypothesis that strong A-tract periodicity and concomitant compaction of the nucleoid could benefit the host by hindering integration of phage DNA into the host chromosome [11].

## Supporting Information

File S1 **Raw data for genomes analyzed in this work.**
(XLS)

File S2 **Raw data for correlations of local NAP binding potential and DNA bends in** ***E. coli*** **.**
(XLSX)

File S3 Cumulative distributions of MaxQ* indices.
(PDF)

File S4 **Comparison of results using the “A2T2” and “AT4” methods.**
(PDF)

File S5 **Data for a set of 25 control genes.**
(PDF)

File S6 **Correlations between the number of predicted DNA bends and the number of predicted NAP binding sites in** ***E. coli*** **.**
(PDF)
