# Supplementary material for: Investigating the Interplay between Nucleoid-Associated Proteins, DNA Curvature, and CRISPR Elements Using Comparative Genomics
Source: PLoS One. 2014 Mar 3;9(3):e90940. doi: 10.1371/journal.pone.0090940 (PMC3940949; doi:10.1371/journal.pone.0090940)
Supplement: File S3 — Cumulative distributions of MaxQ* indices. (PDF) [file pone.0090940.s003.pdf]

Cumulative distributions of MaxQ* indices for genomes with and without each NAP. The ordinate (quantile) shows the fraction of genomes in each group with MaxQ* less or equal to the value shown by the abscissa.

HNS:

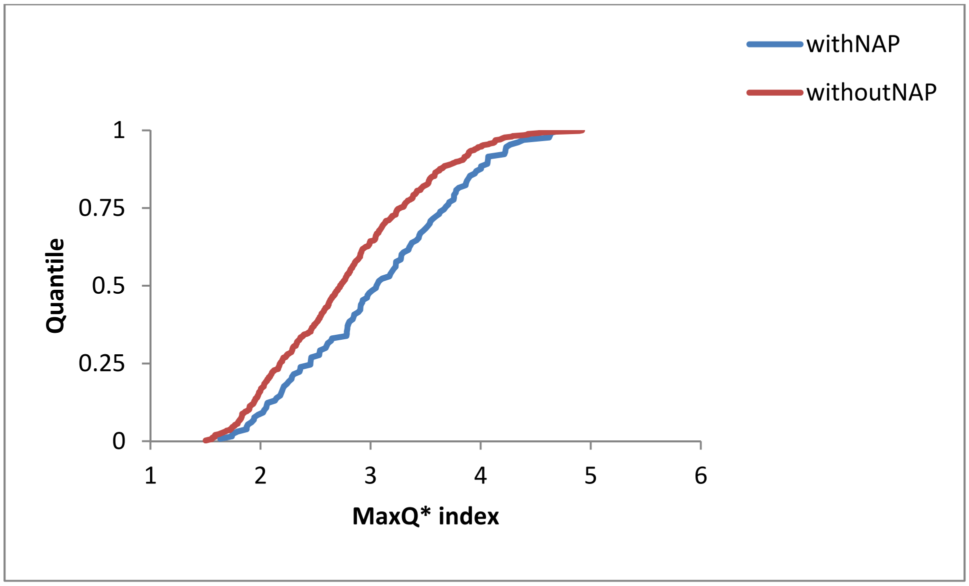

StpA:

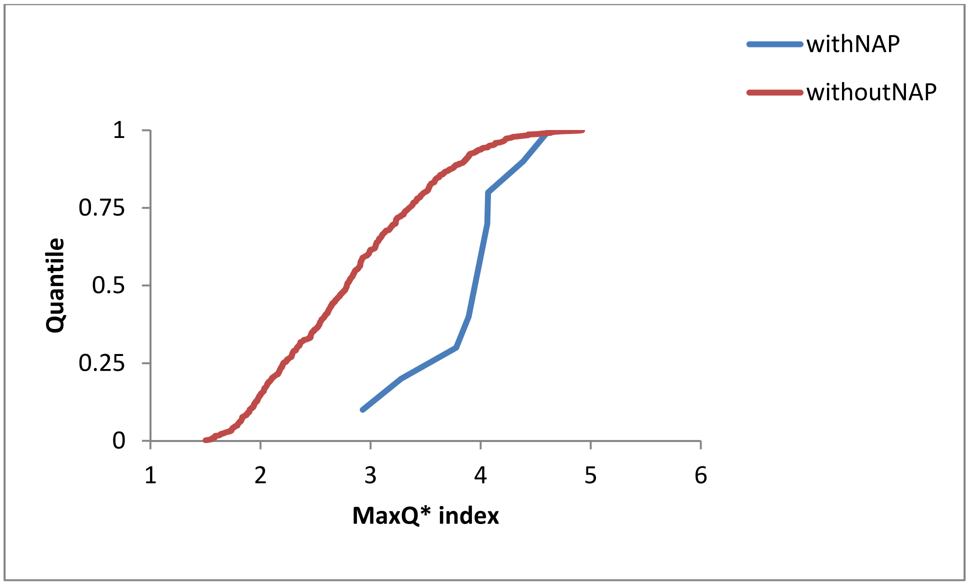

MukB:

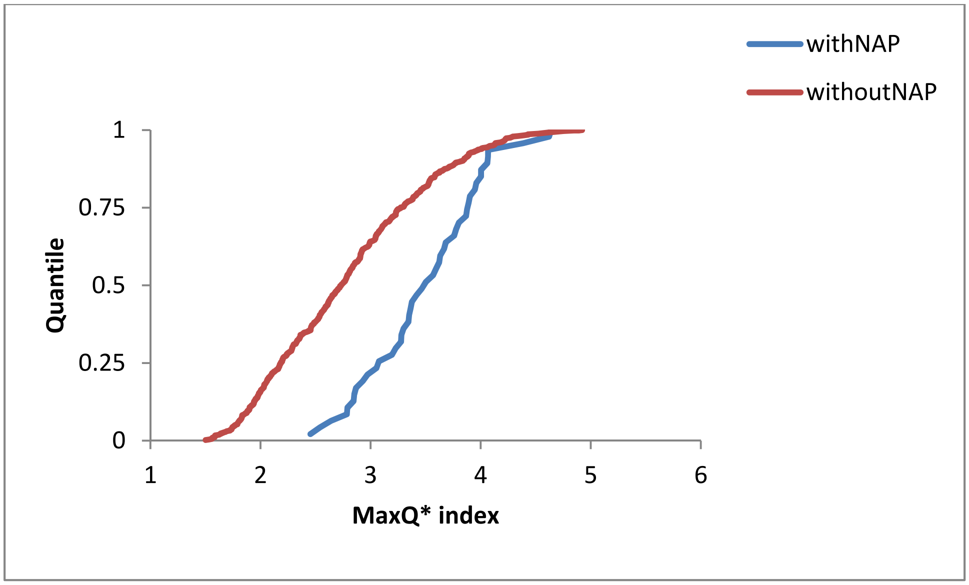

withNAP
withoutNAP
Quantile
1
0.75
0.5
0.25
0
1
2
3
4
5
6
MaxQ* index

Lrp:

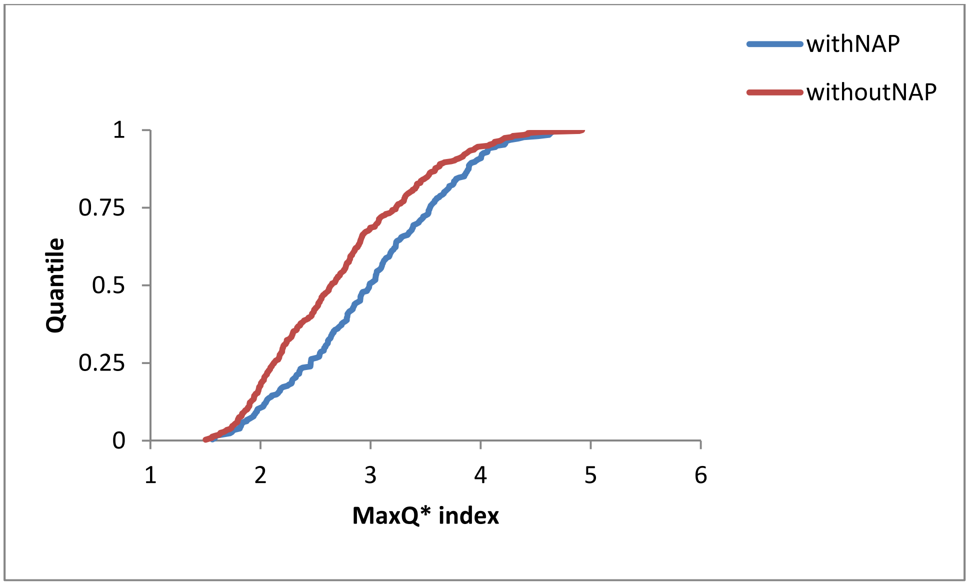

withNAP
withoutNAP
Quantile
1
0.75
0.5
0.25
0
1
2
3
4
5
6
MaxQ* index

Fis:

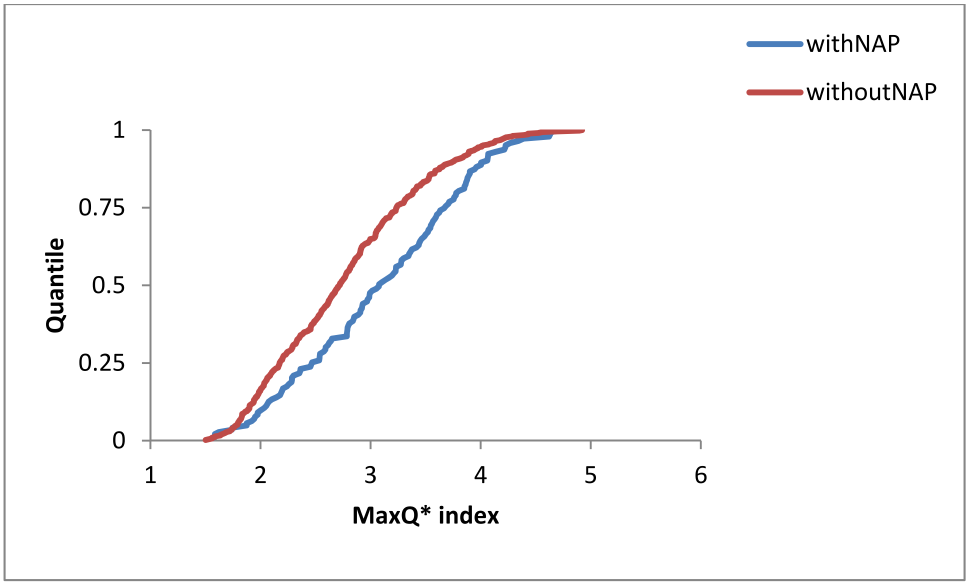

Non-Bridging Protein

IHF-beta:

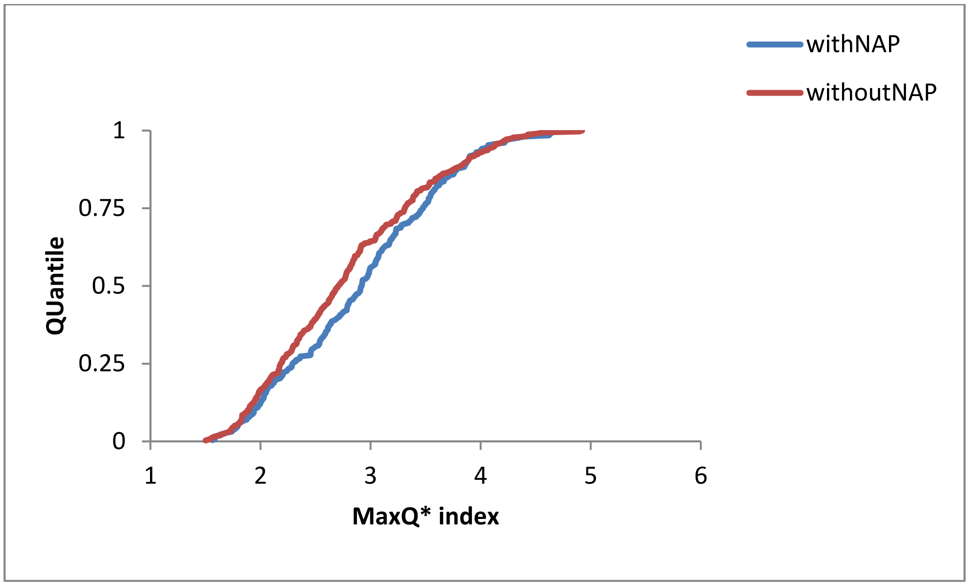

IHF-alpha:

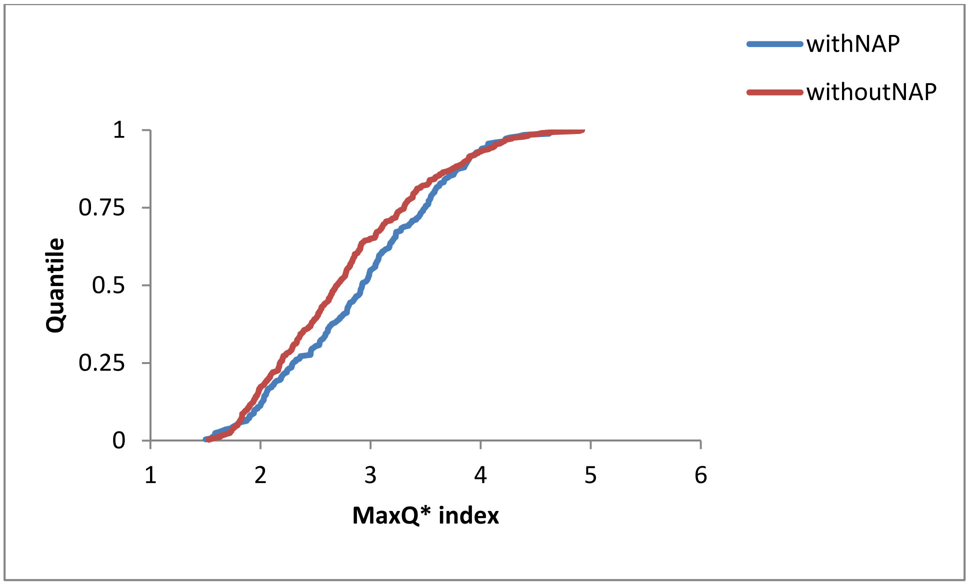

HU-alpha:

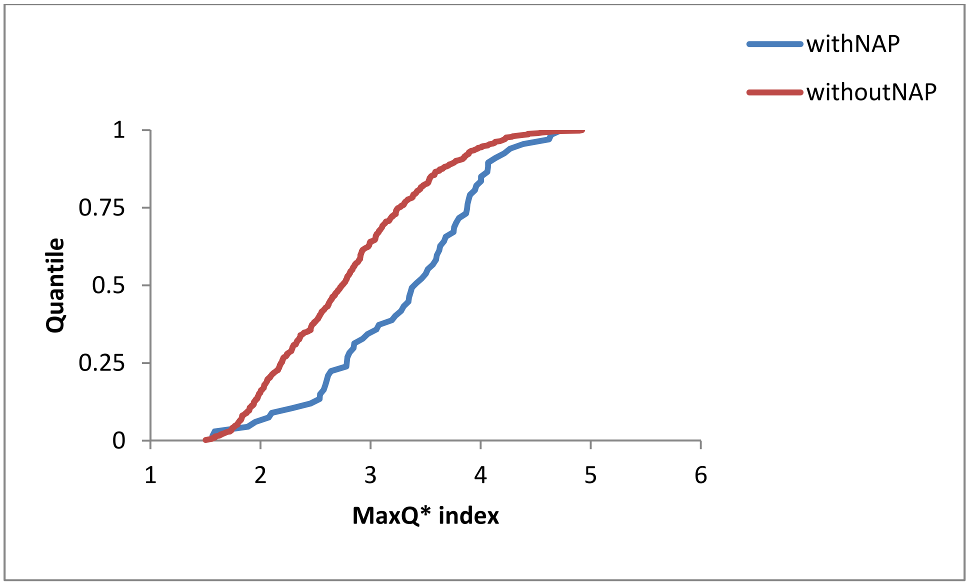

HU-beta:

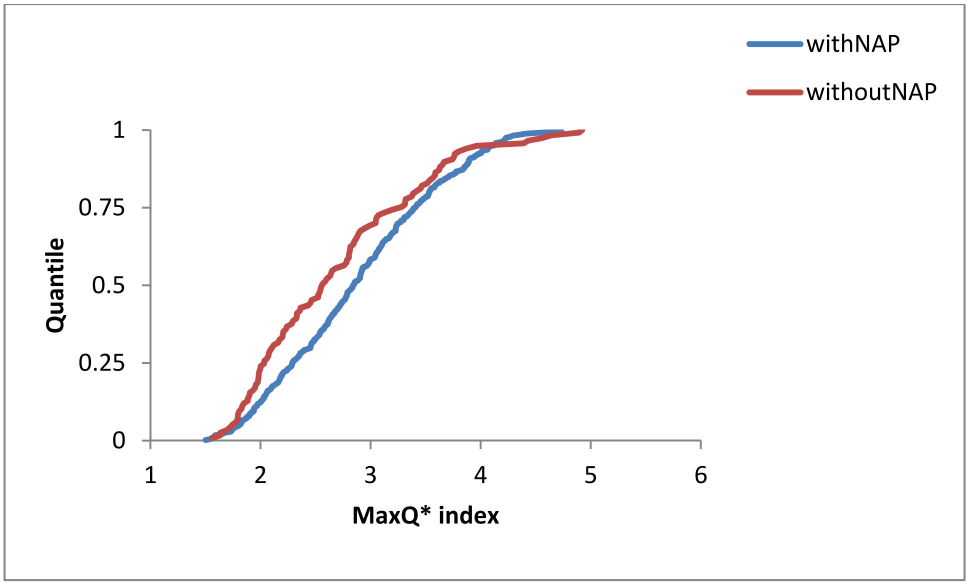
withNAP
withoutNAP
Quantile
1
0.75
0.5
0.25
0
1
2
3
4
5
6
MaxQ* index

Dps:

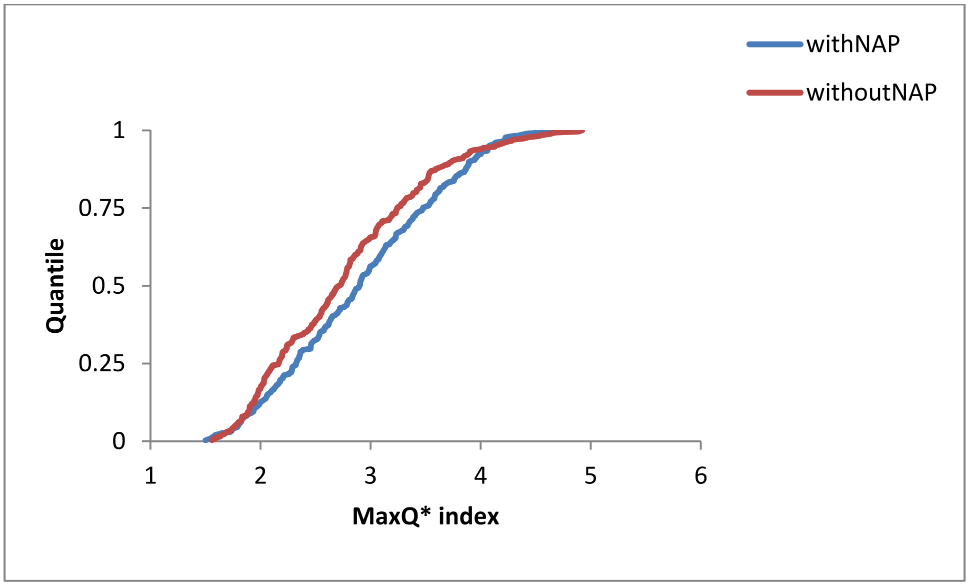
withNAP
withoutNAP
Quantile
1
0.75
0.5
0.25
0
1
2
3
4
5
6
MaxQ* index

CbpA:

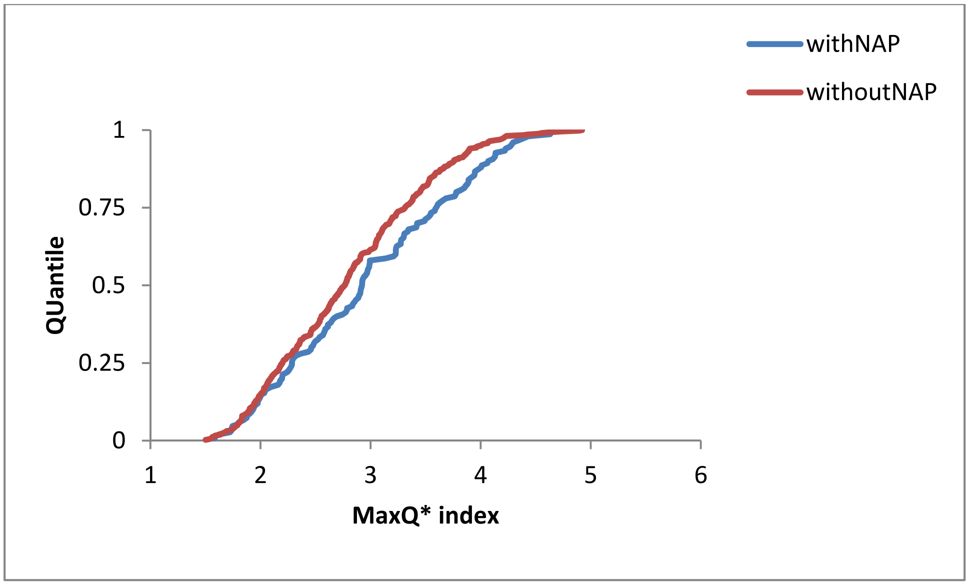
withNAP
withoutNAP
1
0.75
0.5
0.25
0
QUantile
1
2
3
4
5
6
MaxQ* index
