# Supplementary material for: Investigating the Interplay between Nucleoid-Associated Proteins, DNA Curvature, and CRISPR Elements Using Comparative Genomics
Source: PLoS One. 2014 Mar 3;9(3):e90940. doi: 10.1371/journal.pone.0090940 (PMC3940949; doi:10.1371/journal.pone.0090940)
Supplement: File S4 — Comparison of results using the “A2T2” and “AT4” methods. (PDF) [file pone.0090940.s004.pdf]

Table 1. Summary MaxQ statistics in genomes having and lacking specific NAPs in all analyzed genomes

Interaction mode	NAP	"A2T2" method					"AT4" method				
		Genomes with the NAP		Genomes lacking the NAP		*p*-value	Genomes with the NAP		Genomes lacking the NAP		*p*-value
		N	Mean MaxQ ± SD	N	Mean MaxQ ± SD		N	Mean MaxQ ± SD	N	Mean MaxQ ± SD	
Bridging	H-NS	130	3.08 ± 0.76	443	2.78 ± 0.71	$<10^{-4}$	130	2.70 ± 0.71	443	2.54 ± 0.69	0.007
Bridging	StpA[a]	10	3.90 ± 0.49	563	2.83 ± 0.72	$<10^{-4}$	10	3.25 ± 0.63	563	2.57 ± 0.69	0.002
Bridging	MukB[a]	47	3.50 ± 0.52	526	2.79 ± 0.72	$<10^{-12}$	47	3.05 ± 0.58	526	2.54 ± 0.69	$<10^{-6}$
Bridging	Lrp	255	3.00 ± 0.73	318	2.72 ± 0.72	$<10^{-5}$	255	(2.59) ± 0.68	318	(2.57) ± 0.71	0.48
Br+Be[b]	Fis	143	3.08 ± 0.76	430	2.77 ± 0.71	$<10^{-5}$	143	2.74 ± 0.72	430	2.53 ± 0.68	0.001
Bending	IHF-β	256	2.91 ± 0.73	317	2.79 ± 0.74	0.028	256	(2.58) ± 0.68	317	(2.58) ± 0.71	0.61
Bending	IHF-α	250	2.92 ± 0.73	323	2.78 ± 0.73	0.010	250	(2.61) ± 0.67	323	(2.56) ± 0.72	0.14
Bending	HU-α	67	3.32 ± 0.74	506	2.78 ± 0.71	$<10^{-7}$	67	2.97 ± 0.68	506	2.53 ± 0.68	$<10^{-6}$
Bending	HU-β	456	2.88 ± 0.72	117	2.70 ± 0.78	0.006	456	2.61 ± 0.69	117	2.46 ± 0.71	0.013
None[c]	Dps	306	2.91 ± 0.73	267	2.77 ± 0.73	0.009	306	(2.60) ± 0.70	267	(2.55) ± 0.69	0.41
None[c]	CbpA	150	2.98 ± 0.79	423	2.80 ± 0.71	0.019	150	2.70 ± 0.76	423	2.54 ± 0.67	0.043

Mean MaxQ values assessed by the "A2T2" and "AT4" methods and standard deviations are shown for groups of genomes possessing and lacking a homolog of each NAP. N signifies the number of genomes in each group. Presence of absence of each NAP in a genome is based on data from the KEGG database ((Kanehisa, Goto et al. 2004), http://www.genome.jp/linkdb/). 'Interaction mode' specifies whether the NAP forms bridges or bends upon interaction with DNA (adapted from (Dillon and Dorman 2010)). Statistical significance of the differences was assessed by Man-Whitney U-test. Data corresponding to p-values ≥0.05 are in parentheses. DNA-bridging NAPs are in the top part of the table.

[a] The ratio of genomes with and without the NAP is unbalanced (>10 or <0.1).

[b] Fis can form both bridges and bends.

[c] Dps and CbpA have not been confirmed to form bridges or bends.

Table 2. Summary MaxQ statistics in genomes having and lacking specific NAPs in γ-proteobacterial genomes

Interaction mode	NAP	"A2T2" method					"AT4" method				
		Genomes with the NAP		Genomes lacking the NAP		*p*-value	Genomes with the NAP		Genomes lacking the NAP		*p*-value
		N	Mean MaxQ ± SD	N	Mean MaxQ ± SD		N	Mean MaxQ ± SD	N	Mean MaxQ ± SD	
Bridging	H-NS	77	3.39 ± 0.73	31	2.83 ± 0.80	0.002	77	3.03 ± 0.67	31	2.65 ± 0.68	0.012
Bridging	StpA	10	3.90 ± 0.49	98	3.16 ± 0.78	0.002	10	(3.25) ± 0.63	98	(2.89) ± 0.69	0.13
Bridging	MukB	46	3.51 ± 0.52	62	3.02 ± 0.89	0.005	46	3.07 ± 0.56	62	2.81 ± 0.76	0.030
Bridging	Lrp	90	3.38 ± 0.73	18	2.49 ± 0.68	$<10^{-4}$	90	3.01 ± 0.69	18	2.49 ± 0.54	0.004
Br+Be[b]	Fis[a]	99	3.29 ± 0.76	9	2.51 ± 0.75	0.008	99	2.96 ± 0.70	9	2.48 ± 0.48	0.041
Bending	IHF-β[a]	100	3.29 ± 0.76	8	2.43 ± 0.80	0.008	100	2.97 ± 0.69	8	2.37 ± 0.38	0.016
Bending	IHF-α[a]	101	3.30 ± 0.76	7	2.20 ± 0.50	0.001	101	2.97 ± 0.69	7	2.25 ± 0.22	0.006
Bending	HU-α	66	(3.33) ± 0.74	42	(3.07) ± 0.85	0.14	66	(2.97) ± 0.68	42	(2.84) ± 0.71	0.36
Bending	HU-β	92	(3.24) ± 0.78	16	(3.15) ± 0.85	0.67	92	(2.90) ± 0.70	16	(3.04) ± 0.68	0.43
None[c]	Dps	75	3.35 ± 0.77	33	2.97 ± 0.79	0.019	75	(2.99) ± 0.72	33	(2.76) ± 0.62	0.15
None[c]	CbpA	45	(3.14) ± 0.89	63	(3.29) ± 0.71	0.44	45	(2.85) ± 0.72	63	(2.97) ± 0.68	0.40

See legend to Table 1.

[a] The ratio of genomes with and without the NAP is unbalanced (>10 or <0.1).

[b] Fis can form both bridges and bends.

[c] Dps and CbpA have not been confirmed to form bridges or bends.

Table 3. Summary MaxQ statistics in genomes having and lacking specific NAPs in proteobacterial genomes

Interaction mode	NAP	"A2T2" method					"AT4" method				
		Genomes with the NAP		Genomes lacking the NAP		*p*-value	Genomes with the NAP		Genomes lacking the NAP		*p*-value
		N	Mean MaxQ ± SD	N	Mean MaxQ ± SD		N	Mean MaxQ ± SD	N	Mean MaxQ ± SD	
Bridging	H-NS	127	3.07 ± 0.76	143	2.83 ± 0.69	0.02	127	(2.71) ± 0.71	143	(2.61) ± 0.68	0.17
Bridging	StpA[a]	10	3.90 ± 0.49	260	2.91 ± 0.71	$<10^{-4}$	10	3.25 ± 0.63	260	2.64 ± 0.69	0.007
Bridging	MukB	47	3.50 ± 0.52	223	2.83 ± 0.72	$<10^{-7}$	47	3.05 ± 0.58	223	2.58 ± 0.69	$<10^{-5}$
Bridging	Lrp	172	3.08 ± 0.73	98	2.72 ± 0.68	$<10^{-3}$	172	(2.68) ± 0.69	98	(2.63) ± 0.71	0.44
Br+Be[b]	Fis	143	3.08 ± 0.76	127	2.79 ± 0.66	0.002	143	2.74 ± 0.72	127	2.57 ± 0.66	0.05
Bending	IHF-β	237	(2.95) ± 0.71	33	(2.92) ± 0.85	0.95	237	2.61 ± 0.68	33	3.02 ± 0.76	0.005
Bending	IHF-α	243	(2.94) ± 0.72	27	(2.83) ± 0.69	0.4	243	2.61 ± 0.68	27	3.07 ± 0.78	0.004
Bending	HU-α	67	3.32 ± 0.74	203	2.82 ± 0.69	$<10^{-5}$	67	2.97 ± 0.68	203	2.56 ± 0.68	$<10^{-4}$
Bending	HU-β	244	(2.94) ± 0.72	26	(2.99) ± 0.81	0.69	244	(2.66) ± 0.69	26	(2.70) ± 0.76	0.91
None[c]	Dps	178	(2.99) ± 0.75	92	(2.86) ± 0.68	0.16	178	(2.66) ± 0.73	92	(2.66) ± 0.63	0.64
None[c]	CbpA	104	(3.02) ± 0.78	166	(2.90) ± 0.70	0.28	104	(2.75) ± 0.73	166	(2.60) ± 0.68	0.12

See legend to Table 1.

[a] The ratio of genomes with and without the NAP is unbalanced (>10 or <0.1).

[b] Fis can form both bridges and bends.

[c] Dps and CbpA have not been confirmed to form bridges or bends.
