# Supplementary material for: Investigating the Interplay between Nucleoid-Associated Proteins, DNA Curvature, and CRISPR Elements Using Comparative Genomics
Source: PLoS One. 2014 Mar 3;9(3):e90940. doi: 10.1371/journal.pone.0090940 (PMC3940949; doi:10.1371/journal.pone.0090940)
Supplement: File S5 — Data for a set of 25 control genes. (PDF) [file pone.0090940.s005.pdf]

Table 1. Summary of relationships between a set of control genes and MaxQ* for the 573-genome sample, proteobacteria sample and γ-proteobacteria sample.

gene	Description	Direction	p_value1	p_value2	p_value3
aroE	shikimate dehydrogenase	+	0.001	6.31E-07	0.050
leuB	3-isopropylmalate dehydrogenase	+	0.025	0.001	0.048
mmuM	homocysteine S-methyltransferase	+	0.840	0.496	0.344
astA	arginine N-succinyltransferase	+	3.96E-06	0.001	0.013
hisG	ATP phosphoribosyltransferase	+	0.032	0.002	0.170
aspB	aspartate aminotransferase	-	0.015	0.001	0.003
serC	PSAT1; phosphoserine aminotransferase	+	2.15E-06	0.008	NA
tyrB	aromatic-amino-acid transaminase	+	0.010	0.396	0.298
lysC	aspartate kinase	+	0.193	0.069	0.004
ureA	urease subunit gamma	-	0.959	0.058	0.113
paaF	echA; enoyl-CoA hydratase	-	0.040	0.001	0.796
argH	ASL; argininosuccinate lyase	+	0.174	0.019	0.125
hemH	FECH; ferrochelatase	+	0.003	0.483	0.024
paaK	phenylacetate-CoA ligase	-	0.565	0.077	0.658
glnA	glutamine synthetase	+	0.435	0.227	0.217
bioD	dethiobiotin synthetase	+	4.19E-06	0.002	0.123
cobF	precorrin-6A synthase	-	0.416	0.293	0.435
mecR1	methicillin resistance protein	-	0.025	NA	NA
ribB	RIB3; 3,4-dihydroxy 2-butanone 4-phosphate synthase	+	0.004	0.002	0.007
thiC	thiamine biosynthesis protein ThiC	+	0.407	0.514	0.668
moaD	molybdopterin synthase sulfur carrier subunit	+	0.0002	7.29E-06	4.83E-05
cefD	isopenicillin-N epimerase	-	0.982	0.309	NA
acdH	short/branched chain acyl-CoA dehydrogenase	-	0.987	NA	NA
spuC	putrescine aminotransferase	-	0.516	0.061	0.104
nicC	6-hydroxynicotinate 3-monooxygenase	-	0.017	0.003	0.480

'Direction' indicates a positive or negative relationship between presence of the gene and MaxQ* ('+' means that genomes with the gene have higher mean MaxQ* than genomes without the gene); P_value1 refers to the significance of the MaxQ* differences between genomes that possess a homolog of the gene and those where the homolog is absent in the 573-genome sample, p-value2 in the proteobacteria sample, and p-value3 in the γ-proteobacteria sample. NA indicates that statistical tests cannot be performed due to lack of data.
