# Supplementary material for: Investigating the Interplay between Nucleoid-Associated Proteins, DNA Curvature, and CRISPR Elements Using Comparative Genomics
Source: PLoS One. 2014 Mar 3;9(3):e90940. doi: 10.1371/journal.pone.0090940 (PMC3940949; doi:10.1371/journal.pone.0090940)
Supplement: File S6 — Correlations between the number of predicted DNA bends and the number of predicted NAP binding sites in E. coli . (PDF) [file pone.0090940.s006.pdf]

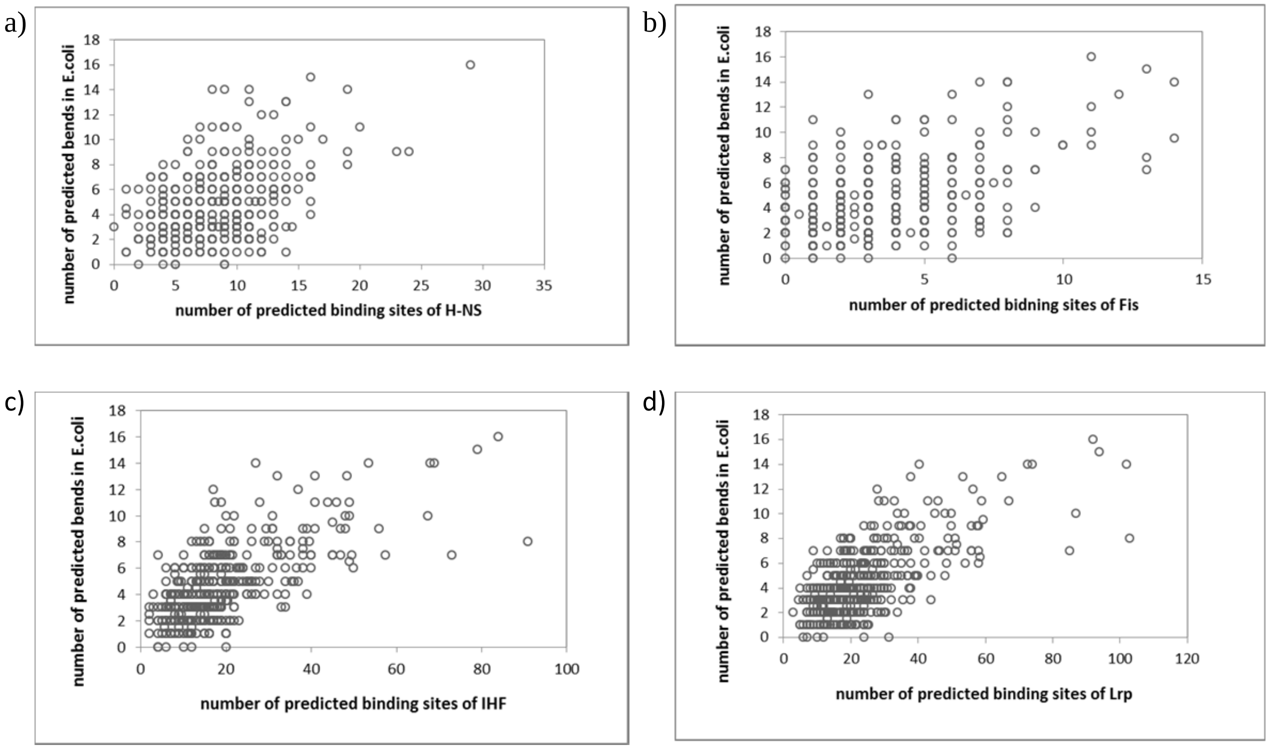

Figure S1 Correlation between the number of predicted DNA bends and the number of predicted binding sites in *E. coli K12* for a) H-NS, b) Fis, c) IHF and d) Lrp with the alternative parameter set.

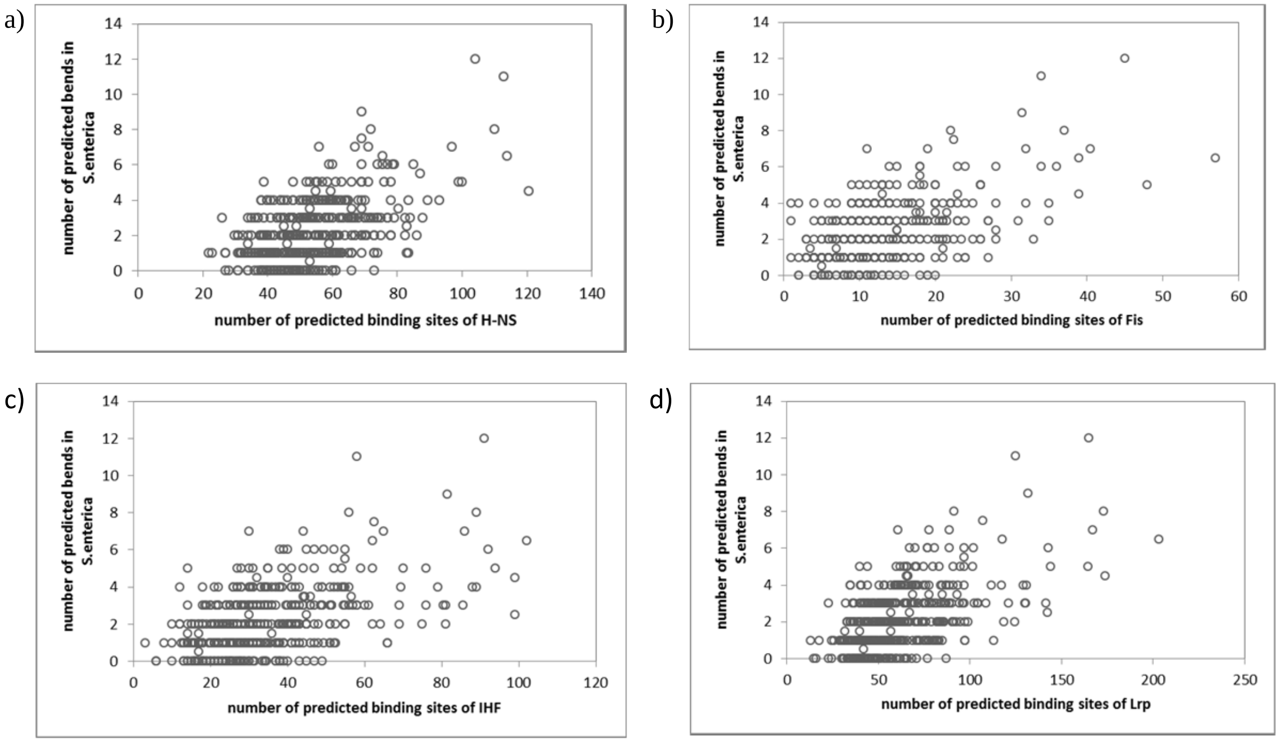

Figure S2 Correlation between the number of predicted DNA bends and the number of predicted binding sites in *S.enterica LT2* for a) H-NS, b) Fis, c) IHF and d) Lrp with the preferred parameter set.

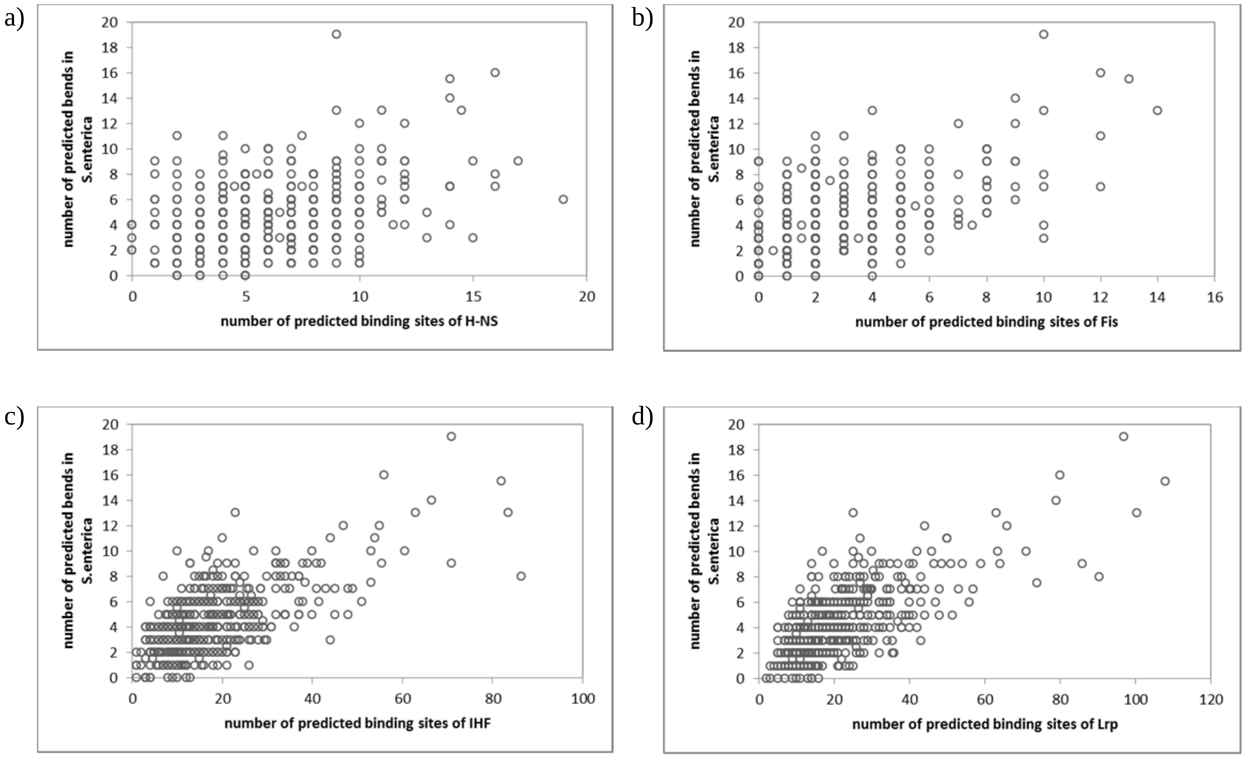

Figure S3 Correlation between the number of predicted DNA bends and the number of predicted binding sites in *S.enterica LT2* for a) H-NS, b) Fis, c) IHF and d) Lrp with the alternative parameter set.
